# Comparative Transcriptomics Uncover the Uniqueness of Oocyte Development in the Donkey

**DOI:** 10.3389/fgene.2022.839207

**Published:** 2022-01-28

**Authors:** Fa-Li Zhang, Shu-Er Zhang, Yu-Jiang Sun, Jun-Jie Wang, Wei Shen

**Affiliations:** ^1^ College of Life Sciences, Key Laboratory of Animal Reproduction and Biotechnology in Universities of Shandong, Qingdao Agricultural University, Qingdao, China; ^2^ College of Animal Science and Veterinary Medicine, Shandong Agricultural University, Tai’an, China; ^3^ Animal Husbandry General Station of Shandong Province, Jinan, China; ^4^ Dongying Vocational Institute, Dongying, China

**Keywords:** donkey, oocyte development, WGCNA, comparative transcriptomic, WEE2

## Abstract

The donkey is an important domestic animal, however the number of donkeys world-wide is currently declining. It is therefore important to protect their genetic resources and to elaborate the regulatory mechanisms of donkey reproduction, particularly, oocyte development. Here, we adopted comparative transcriptomic analysis and weighted gene co-expression network analysis (WGCNA) to uncover the uniqueness of donkey oocyte development compared to cattle, sheep, pigs, and mice, during the period from germinal vesicle (GV) to metaphase II (MII). Significantly, we selected 36 hub genes related to donkey oocyte development, including wee1-like protein kinase 2 (WEE2). Gene Ontology (GO) analysis suggested that these genes are involved in the negative regulation of cell development. Interestingly, we found that donkey specific differentially expressed genes (DEGs) were involved in RNA metabolism and apoptosis. Moreover, the results of WGCNA showed species-specific gene expression patterns. We conclude that, compared to other species, donkey oocytes express a large number of genes related to RNA metabolism to maintain normal oocyte development during the period from GV to MII.

## Introduction

The donkey (*Equus asinus*) is a descendent of the African wild ass and is a common domestic beast of burden (Beja-Pereira et al., 2004). It can also provide meat and milk, and in particular donkey-hide gelatin, mostly composed of collagen, which is a traditional Chinese medicinal material (Kim et al., 2018). There is a broad consensus that, owing to the high content of linoleic acid, donkey meat is very palatable (Polidori et al., 2009). However, it is worrying that the number of donkeys is currently declining sharply owing to agricultural mechanization and the development of transportation vehicles.

Animal reproductive biotechnology, including *in vitro* fertilization and embryo transfer (IVF-ET) and its derivative technologies, is a perfect technology for saving endangered species (Bai et al., 2020). However, a limitation of this technology with donkeys is that the rate of *in vitro* oocyte maturation is relatively low (Goudet et al., 2016). Therefore, a deeper understanding of the characteristics of donkey oocyte development is needed, focusing on the meiotic maturation of oocytes, during the period from germinal vesicle (GV) to metaphase II (MII). The period from GV–MII marks the maturation of an oocyte, both nuclear and cytoplasmic (Eppig, 1996; Zhao et al., 2020). Studying the core regulatory factors of oocyte development during this period, and the precise and meticulous regulatory signaling pathways are of great significance for improving the *in vitro* maturation rate of donkey oocytes.

Previous studies mainly focused on economic traits, comparing donkeys with other livestock, but little research has addressed gene expression characteristics (Aganga et al., 2003; Polidori et al., 2008; Tian et al., 2020). Currently, research regarding single-cell RNA-seq (scRNA-seq) data of donkey oocytes suggests that the differentially expressed genes (DEGs) during GV–MII are related to the meiotic cell cycle, mitochondrial activity, and so on (Li Z. et al., 2021). The research suggested that wee1-like protein kinase 2 (WEE2) was involved in donkey oocyte development. Moreover, a study in mice indicated that Wee2-deficient caused fertilization failure and female infertility (Sang et al., 2018). WEE2 is indispensable for ensuring exit from meiosis in oocytes and promote pronuclear formation (Oh et al., 2011). However, the function of WEE2 is still unclear so far. In 2013, the donkey genome was been reported for the first time by Orlando et al., and in 2018, they released an updated version (Orlando et al., 2013; Renaud et al., 2018). In 2020, the Dezhou donkey genome was reported by Wang et al. (Wang C. et al., 2020). The reports of these reference genomes provide us with convenient conditions for studying the gene expression characteristics of donkey oocyte development.

RNA sequencing (RNA-seq) is able to determine the sum of all transcribed RNAs of tissues or cells under specific conditions, and has been widely used in animal and plant research (Stark et al., 2019). However, the limitation of this technology is that it requires large number of cells, which is not ideal for precious cell types such as oocytes. Excitingly, the emergence of scRNA-seq solves the problem of how to obtain the transcriptome status of a very small number of cells (Picelli et al., 2013). Here, we collected GV phase and MII phase scRNA-seq data from donkeys, cattle, sheep, pigs, and mice, and applied comparative transcriptomics analysis and weighted gene co-expression network analysis (WGCNA). Surprisingly, including WEE2, we identified 36 hub genes related to donkey oocyte development during GV–MII stages. This study provides novel information about key regulators of donkey oocyte development, which can provide a theoretical basis for the protection of donkey germplasm resources.

## Materials and Methods

### Data Collection

We collected transcriptome data of oocyte development during the period from germinal vesicle (GV) to metaphase II (MII) in different species, including donkeys, cattle, sheep, pigs, and mice. All RNA sequencing (RNA-seq) data was derived from public databases, including sequence read archive (SRA), gene expression omnibus (GEO), and genome sequence archive (GSA) with the following accession numbers: donkey (PRJNA763991) (Li Z. et al., 2021), cattle (CRA005589) (Li M.-H. et al., 2021), pig (GSE160334) (Du et al., 2021), sheep (GSE148022) (Wang J.-J. et al., 2020), and mouse (GSE119906) (Qian et al., 2019).

### Workflow of RNA-Seq Data Processing

In order to ensure the accuracy of data analysis, we dealt with raw sequencing data, not processed files. Firstly, the FastQC (v0.11.8) was used to check the raw RNA-seq data and based on the quality control report, unqualified data was eliminated (Andrews, 2010). Next, Fastp (v0.23.1) was used for further quality control, and in this step, low-quality, unqualified reads were removed (Chen et al., 2018). As important sequence alignment software, STAR (v2.7.0f) was selected for sequence alignment to a reference genome, and we directly used the “*--outSAMtype BAM SortedByCoordinate*” parameter to generate a BAM format file (Dobin et al., 2013). The reference genome of each species was as follows: *Equus asinus* (assembly ASM1607732v2), *Bos taurus* (assembly ARS-UCD1.2), *Ovis aries* (assembly ARS-UI_Ramb_v2.0), *Sus scrofa* (assembly Sscrofa11.1), and *Mus musculus* (assembly GRCm38.p5). Finally, FeatureCounts (v1.6.3) was processed to generate gene counts (Liao et al., 2014).

### Differentially Expressed Genes Analysis

The R package DESeq2 (v1.32.0) was adopted to identify the DEGs of oocytes during GV–MII (Love et al., 2014). The judgment threshold of significantly different DEGs was “|log2fold change| > 2 and *p*-value < 0.05”. In order to quantify the amount of gene expression, we used a custom R script to calculate fragments per kilobase of exon model per million mapped fragments (FPKM) (Zhao et al., 2021).

### Comparative Analysis of Oocyte Development Among Species

To obtain a comprehensive understanding of the uniqueness of donkey oocyte development during GV–MII, compared to other species, we used comparative transcriptomic analysis. Firstly, we used uniform conditions to obtain DEGs during GV–MII. Next, taking gene function annotations into consideration, the gene symbols in donkeys, cattle, sheep, and pigs, were converted into murine homologous gene symbol IDs via the R package gprofiler2 (v0.2.1) (Kolberg et al., 2020). Finally, we compared the similarities and differences between donkey and cattle, sheep, pig, and mouse oocyte development during GV–MII.

### Principal Component Analysis (PCA) and Hierarchical Clustering Analysis

PCA of gene expression counts in this study were performed using the R package DESeq2 (v1.32.0), and the top 2 PCs were displayed by scatter plot (Love et al., 2014). The FPKM matrix of gene expression in the examined species, underwent hierarchical clustering analysis and the R package ggtree (v3.0.4) was used for visualization (Yu et al., 2017).

### Weighted Gene Co-expression Network Analysis (WGCNA)

The R package WGCNA (v1.70-3) was used to uncover the correlation between genes (Langfelder and Horvath, 2008). First, FPKM was used to normalize gene expression levels among all species in the study. Next, in order to construct a mixed matrix of the gene expression of different species, the R package gprofiler2 (v0.2.1) was selected to unify the gene symbol IDs (Kolberg et al., 2020). Further, the function *pickSoftThreshold*() of R package WGCNA to ensure a scale-free network, the soft threshold of *β* was set to 16. A hierarchical clustering dendrogram, a heat map, and a topology overlap matrix (TOM) were used to show the relationship between the functional modules and genes. Cytoscape software (v3.8.2) was used to exhibit the top 10 topological overlap relationships in functional modules related to donkey oocyte development during GV–MII.

### Identification of Hub Genes and Protein-Protein Interaction Network Analysis

The hub genes, referring to highly interconnected nodes in functional modules, are considered as functionally important genes in WGCNA (Langfelder and Horvath, 2008). The hub genes were selected by the module membership and gene significance (MM & GS) method, and the conditional threshold was set as MM > 0.98 and SG > 0.8. Next, the hub genes were placed into the PPI network analysis through STRING (v11.5) (https://string-db.org/) to observe the interaction between genes.

### Gene Ontology and Kyoto Encyclopedia of Genes and Genomes Analysis

DEGs were processed for GO and KEGG analysis using clusterProfiler (v4.0.5) (Wu et al., 2021). Considering that different function annotation libraries may lead to differences in results, Metascape, a web-based and timeously updated biological annotation database was also used for the same analysis of hub genes (Zhou et al., 2019).

## Results

### Overview of the Transcriptome Landscape of Oocytes in Different Species

**FIGURE 1:**
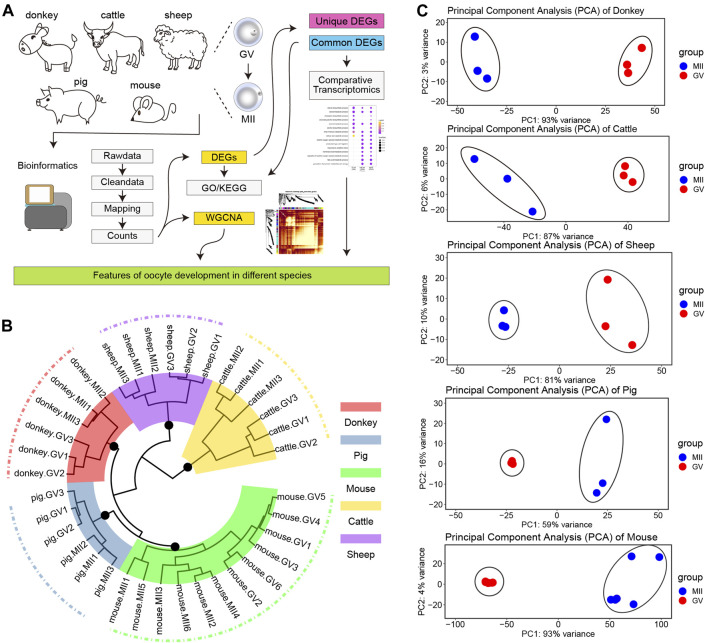
Transcriptome landscape of oocyte development in different species. **(A)** The experimental design flow chart of this study. **(B)** Hierarchical clustering of the transcriptome landscape of oocyte development in different species; different colors represent different species: red, donkey; steel blue, pig; green, mouse; gold, cattle; and purple, sheep **(C)** Principal component analysis (PCA) of different species; from top to bottom: donkey, cattle, sheep, pig, and mouse.

Committed to obtaining key regulatory factors and signal pathways for donkey oocyte development, we designed the experimental program shown in Figure 1A. First, we collected the single cell RNA sequencing data of GV and MII phase oocytes in the studied species (the detail information in Supplementary Table S1). To achieve improved analysis results, we took a strict bioinformatics analysis process (as described in Materials and Methods). Next, we focused on the DEGs of the different species, and adopted a plan of pairwise comparison and overall comprehensive comparison. Moreover, we carried out WGCNA of all uniformed detected genes of oocyte development during GV–MII in different species (Figure 1A). Unsurprisingly, it uncovered that the transcriptome profile of oocyte development during GV–MII between different species was species-specific, and in the same species, the hierarchical clustering indicated that there was a clear difference between GV and MII stages (Figure 1B). Furthermore, in all species, the GV and MII stages showed a clear dividing line, which indicated that the transcription characteristics of these two periods were clearly separated (Figure 1C).

### Identification of the DEGs in Oocytes From GV to MII in Different Species

**FIGURE 2:**
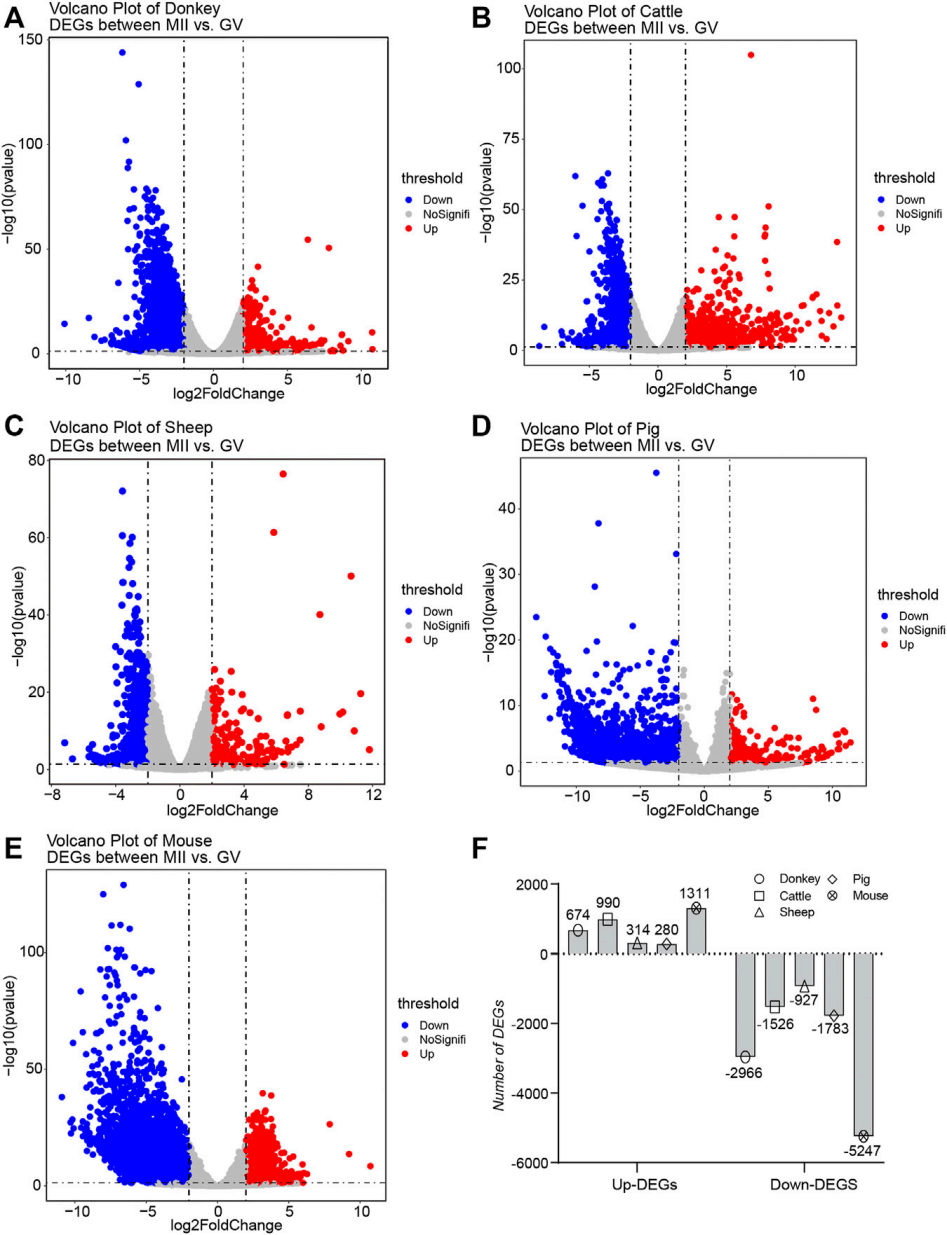
Overview of the differentially expressed genes (DEGs) during oocyte development in different species **(A–E)** The volcano plot exhibits the differential expression levels of oocytes during the period from germinal vesicle (GV) to metaphase II (MII) in different species, including donkey, cattle, sheep, pig, and mouse. **(F)** The bar plot shows the number of DEGs during the period from GV to MII in different species.

There is a broad consensus that DEGs tend to serve as key factors with the potential to regulate the transformation of cell fate (Stark et al., 2019; Zhang et al., 2019). Consequently, we collected detailed statistics of all the DEGs in oocyte development during GV–MII in different species. For the donkey, the number of down-regulation DEGs (down-DEGs) was 2,966, and the number of up-DEGs was 674 (Figure 2A and detail in Supplementary Table S2). For cattle, the number of down-DEGs was 1,526, and the number of up-DEGs was 990 (Figure 2B and detail in Supplementary Table S3). For sheep, the number of down-DEGs was 927, and the number of up-DEGs was 314 (Figure 2C and detail in Supplementary Table S4). For pigs, the number of down-DEGs was 1783, and the number of up-DEGs was 280 (Figure 2D and detail in Supplementary Table S5). For mice, the number of down-DEGs was 5,247, and the number of up-DEGs was 1,311 (Figure 2E and detail in Supplementary Table S6). In general, the number of down-DEGs was much greater than the number of up-DEGs; moreover, the number of DEGs detected in sheep was the least among all species (Figure 2F).

### Difference of Oocyte Development in the Donkey Compared With Other Species

**FIGURE 3:**
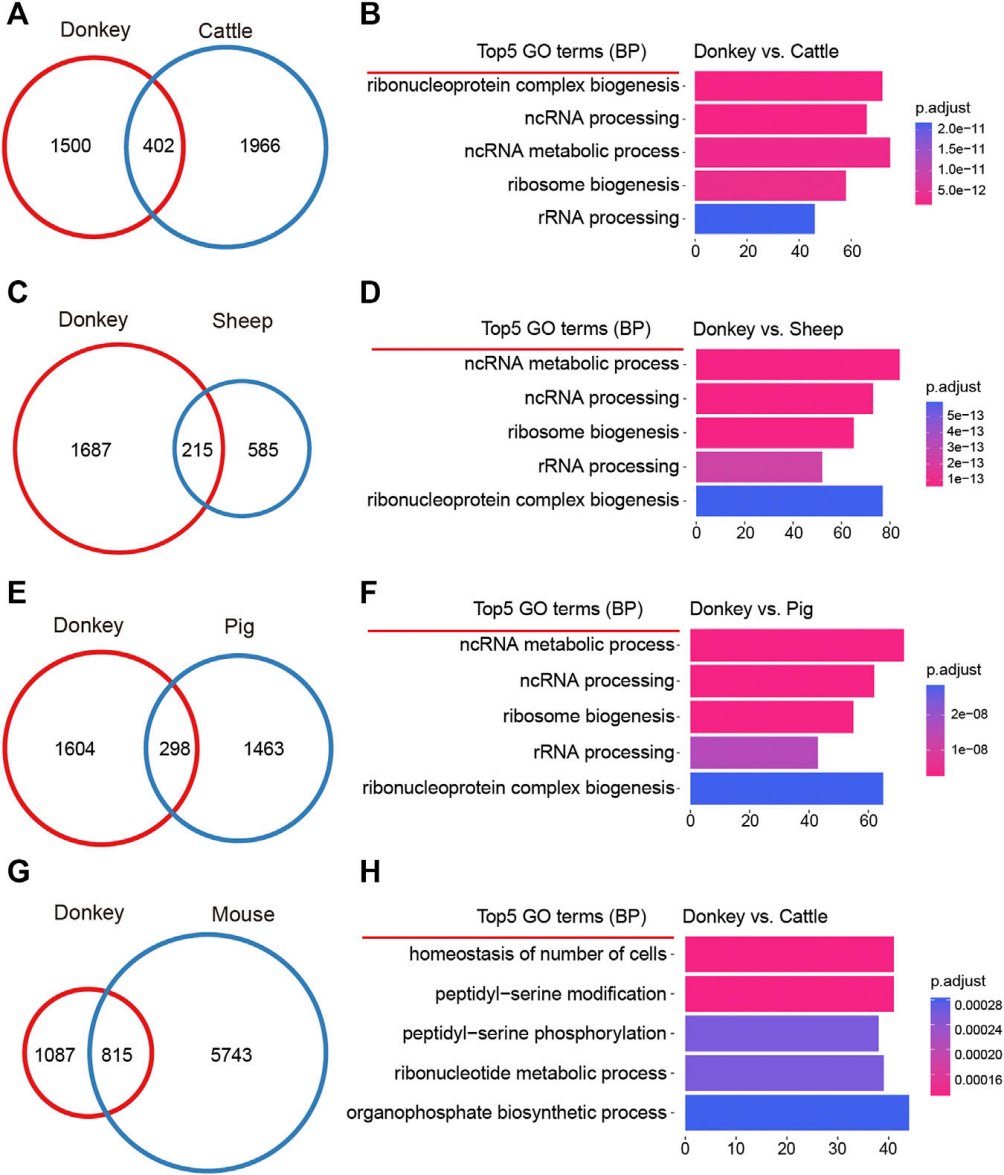
Comparative analysis of donkey oocyte development during the period from germinal vesicle (GV) to metaphase II (MII). **(A)** The differences in oocyte development between donkeys and cattle during GV–MII. **(B)** The top5 GO (biological process) terms of donkey unique differentially expressed genes (DEGs) compared to cattle. **(C)** The differences in oocyte development between donkeys and sheep during GV–MII. **(D)** The top5 GO (biological process) terms of donkey unique DEGs compared to sheep. **(E)** The differences in oocyte development between donkeys and pigs during GV–MII. **(F)** The top5 GO (biological process) terms of donkey unique DEGs compared to pigs. **(G)** The differences in oocyte development between donkeys and mice during GV–MII. **(H)** The top5 GO (biological process) terms of donkey unique DEGs compared to mice.

Organisms of different species have species-specific development (Yamashita et al., 2000); we may therefore ask whether oocyte development in different species also has species-specificity? To address this question, we made a detailed comparison of DEGs during oocyte development in donkeys and other species. For donkeys and cattle, when comparing the DEGs of oocyte development during GV–MII, we found a total of 402 identical DEGs; and for donkeys, there were 1,500 donkey specific DEGs (Figure 3A). On the 1,500 donkey specific DEGs, we performed GO analysis, and found that the top5 terms focused on non-coding RNA (ncRNA) metabolic process (Figure 3B). For donkeys and sheep, we found a total of 215 identical DEGs, and 1,687 donkey specific DEGs (Figure 3C). GO analysis of the 1,687 donkey specific DEGs found that the top5 terms focused on the ncRNA metabolic process (Figure 3D). For donkeys and pigs, we found a total of 298 identical DEGs, and 1,604 donkey specific DEGs (Figure 3E) of which GO analysis indicated that the top5 terms focused on the ncRNA metabolic process (Figure 3F). For donkeys and mice, we found 815 identical DEGs, with 1,087 of donkey specific DEGs (Figure 3G) of which GO analysis, indicated that the top5 terms included the homeostasis of number of cells (Figure 3H).

### The Uniqueness of Donkey Oocyte Development During GV–MII

**FIGURE 4:**
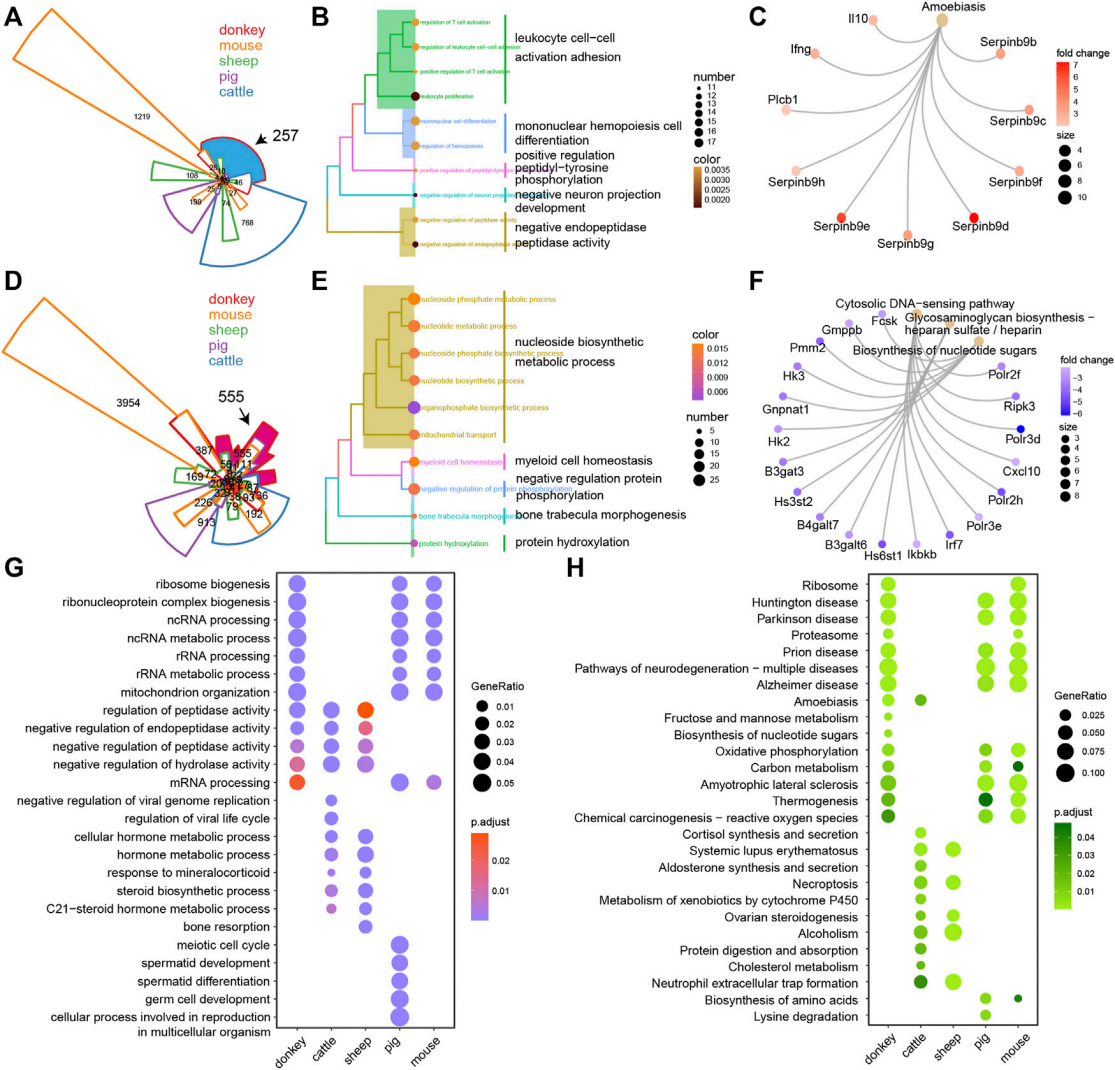
The unique transcriptional characteristics of donkey oocyte development during the period from germinal vesicle (GV) to metaphase II (MII). **(A)** Venn plot of the up-regulated differentially expressed genes (up-DEGs) in different species during GV–MII. The black arrow refers to the donkey-specific up-DEGs compared to other species. The area size represents the number of genes, and the area marked in blue contains the donkey-specific up-DEGs. **(B** and **C)** The GO (biological process) terms and KEGG annotation of donkey-specific up-DEGs, respectively. **(D)** Venn plot of the down-DEGs in different species during GV–MII. The black arrow refers to the donkey-specific down-DEGs compared to other species. The area size represents the number of genes, and the area marked in rose red indicates the donkey-specific down-DEGs. **(E** and **F)** The GO (biological process) terms and KEGG annotation of the donkey-specific down-DEGs, respectively. **(G** and **H)** The dot plot shows the GO (biological process) terms and KEGG annotation of the DEGs of donkeys and other species from GV–MII, respectively.

In order to better understand the intricacies of donkey oocyte development, we comprehensively analyzed the similarities and differences of DEGs in oocyte development between the studied species. For up-DEGs, compared with all other tested species, there were 257 donkey-specific genes (Figure 4A). Furthermore, results of GO and KEGG analysis suggested that the 257 donkey specific up-DEGs were involved in cell differentiation and immune process (Figures 4B,C). Interleukin 10 (IL10) is a cytokine involved in inflammation and immunosuppression, and is engaged in the regulation of cell growth and differentiation. Moreover, it participates in apoptosis (Dhingra et al., 2009). Interestingly, we found that IL-10 was a donkey specific up-DEG (Figure 4C). For down-DEGs, there were 555 donkey specific genes (Figure 4D). GO and KEGG analysis indicated that the 555 donkey specific down-DEGs participated in nucleoside biosynthetic metabolic process and cytosolic DNA-sensing pathway (Figures 4E,F). To our surprise, we found that C-X-C motif chemokine 10 (CXCL10) was a donkey specific down-DEG (Figure 4F). Because CXCL10 is an inflammatory cytokine and involved in apoptosis, it stands to reason that these donkey specific down-DEGs are also involved in apoptosis. Moreover, we constructed a list of DEGs of all detected species, including up-DEGs and down-DEGs, and used clusterProfiler’s *compareCluster* function to perform GO and KEGG function analysis on them. For GO analysis, compared with other species, the donkey was mainly reflected in the process of RNA metabolism regulation (Figure 4G). For KEGG analysis, not surprisingly, compared with other species, donkeys had significant enrichment in their ribosomal regulation process, which was well combined with the ncRNA and rRNA metabolism annotation in GO terms (Figure 4H).

### WGCNA of Oocyte Development in Different Species

**FIGURE 5:**
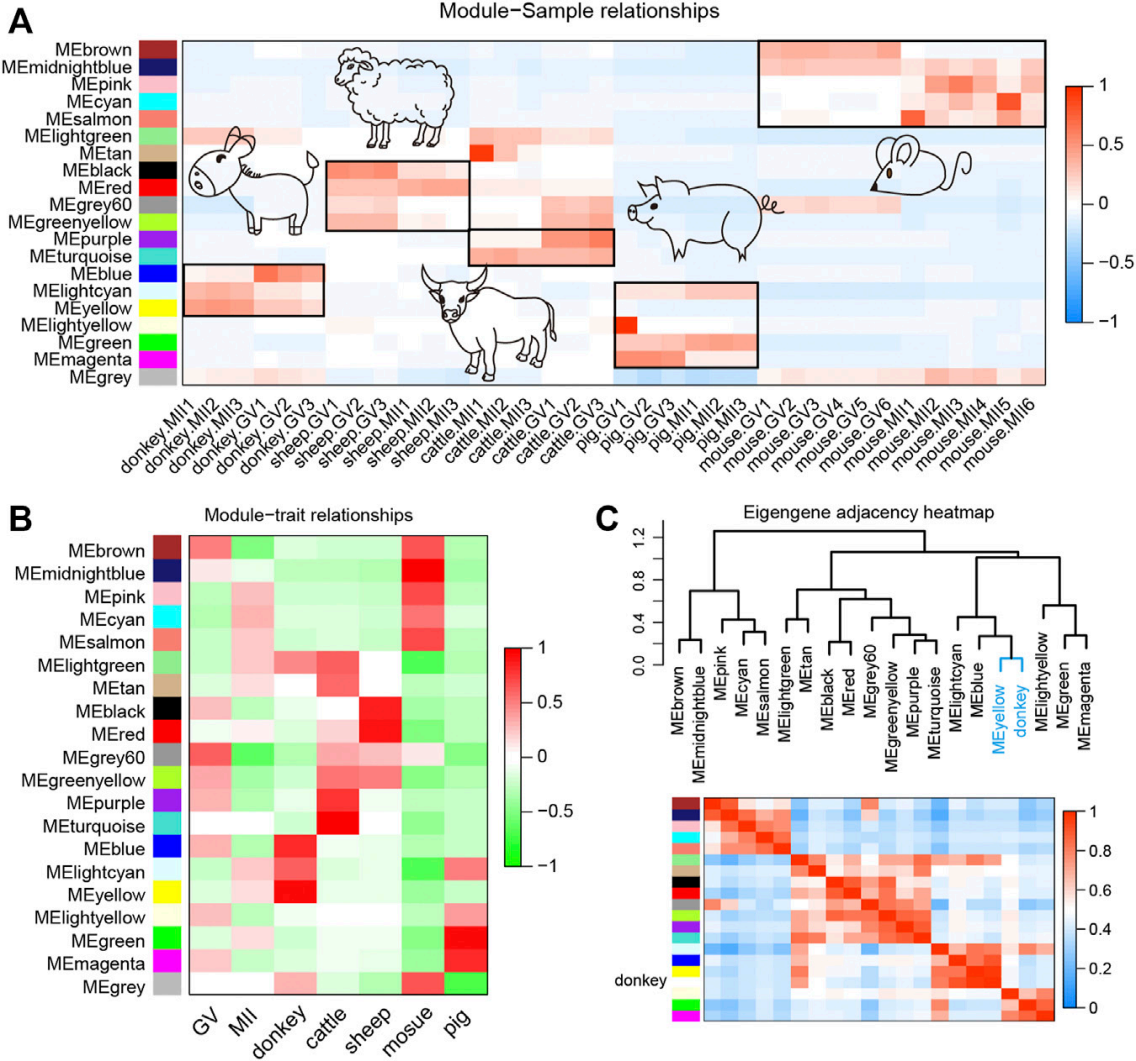
Weighted gene co-expression network analysis (WGCNA) of oocyte development during the period from germinal vesicle (GV) to metaphase II (MII) in different species. **(A)** The heat map shows the correlation between different samples and functional modules identified by WGCNA. **(B)** The heatmap shows the correlation between different phenotypes and functional modules identified by WGCNA. **(C)** The correlation between donkey and functional modules identified by WGCNA. The yellow functional module is the key functional module involved in donkey oocyte development.

WGCNA, increasingly used in bioinformatics analysis, is able to accurately pick out the hub genes related to traits of interest in a complex gene expression profile (Pei et al., 2017). In order to make the correlation between genes conform to the scale-free network distribution, the appropriate soft threshold must be set correctly. Here, we set the soft threshold at 16 to assure the downstream analysis of WGCNA (Supplementary Figure S1A). Next, 20 gene functional modules were identified and exhibited as a hierarchical clustering dendrogram and a module eigengene adjacency heat map (Supplementary Figure S1B). The turquoise module contained the greatest number of genes (Supplementary Figure S1B). Moreover, the heat map of module-sample relationships elucidated that the different functional modules were species-specific, and donkey-specific functional modules were yellow, blue, and light cyan (Figure 5A). The heat map of module-trait relationships also suggested that the different functional modules were species-specific, and donkey-specific functional modules were yellow, blue, and light cyan (Figure 5B). In order to identify the core functional modules that regulate the development of donkey oocytes, we constructed a hybrid matrix pool between the traits of donkeys and functional modules, and performed cluster analysis. The results showed that the yellow modules were closely related to the development of donkey oocytes (Figure 5C).

### Identification of Hub Genes Involved in Donkey Oocyte Development

**FIGURE 6:**
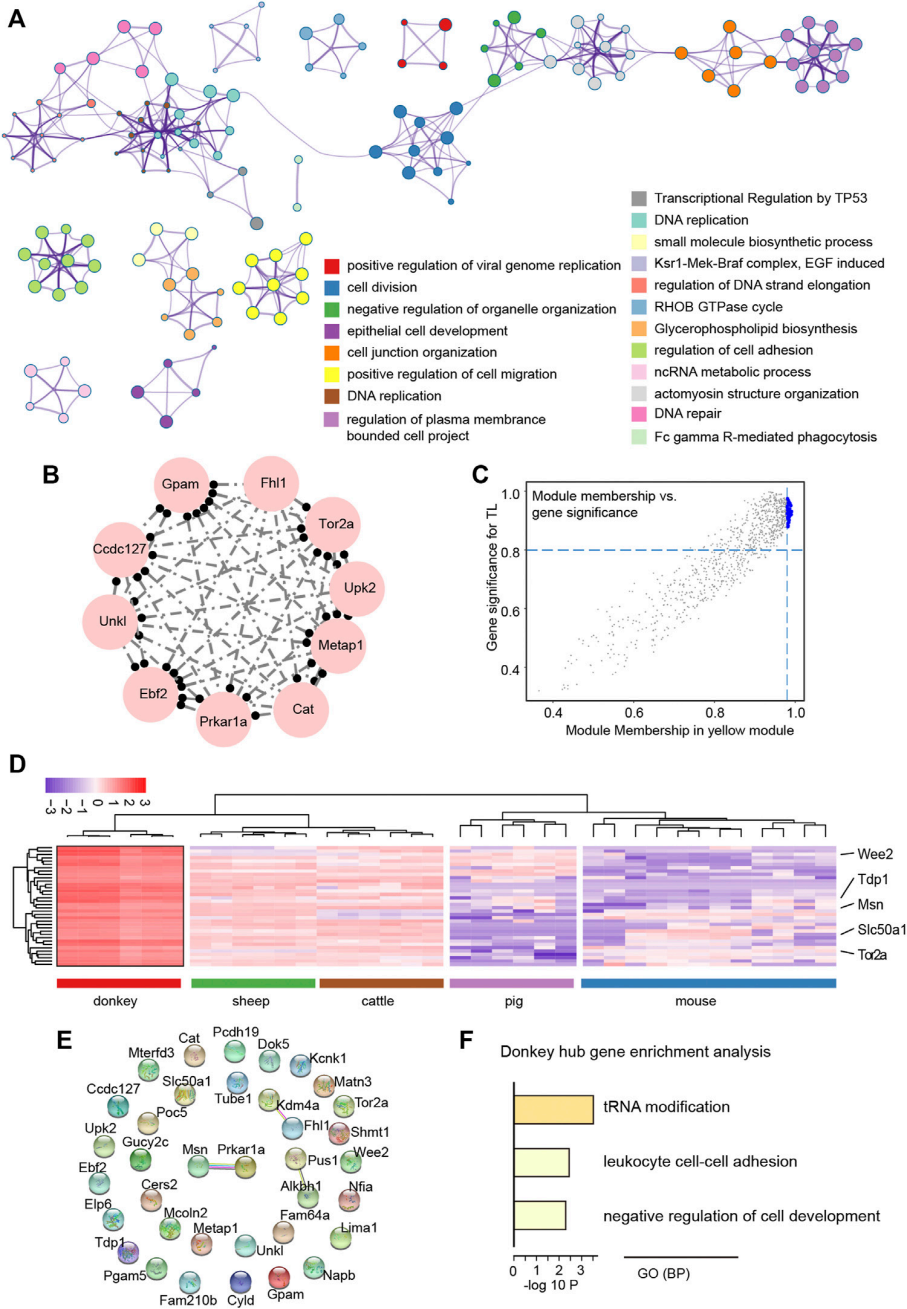
Functional exploration of hub genes related to donkey oocyte development identified by weighted gene co-expression network analysis (WGCNA). **(A)** The GO and KEGG annotation of genes in the yellow functional module. **(B)** The top 10 topological overlap relationship in the yellow functional module. **(C)** The scatter plot shows the relationship between module membership and gene significance (MM & GS). The large blue dots represent hub genes related to donkey oocyte development. **(D)** The heat map shows the expression levels of hub genes selected by the yellow functional module. **(E)** The protein-protein interaction network of hub genes. The connection between proteins represents the degree of protein interaction. **(F)** The GO (biological process) terms of hub genes related to donkey oocyte development.

The TOM plot indicated the interactions between gene functional modules, and the results revealed an obvious strong interactive relationship between different functional modules, which was related to the species specificity between functional modules (Supplementary Figure S1C). In order to further explore the regulatory relationship between genes in the yellow functional module, first the GO analysis of genes in the yellow functional module showed that these genes were involved in meiotic cell division such as DNA repair, and apoptosis process such as transcriptional regulation by the TP53 pathway (Figure 6A). Moreover, the top 10 genes with the strongest topology overlap relationship were exhibited as a circle graph (Figure 6B). Notably, the hub genes were selected by the MM & GS method (Figure 6C); finally, we obtained a total of 36 hub genes. After observing the expression characteristics of these hub genes in different species, we found that these genes, including wee1-like protein kinase 2 (WEE2) that is a notable meiotic gene (Hanna et al., 2010), were highly expressed in donkey oocytes, compared to other species (Figure 6D). In order to systematically understand the interaction of these hub genes, protein-protein interaction (PPI) network analysis showed that the proteins translated by these hub genes did not directly interact strongly, which suggested that these hub genes were involved in different biological pathways to regulate the specificity of donkey oocyte development (Figure 6E). Furthermore, GO analysis showed that these hub genes were related to the negative regulation of cell development (Figure 6F).

## Discussion

Donkey (*Equus asinus*), was domesticated around 3000 B.C. and became a traditional domestic animal whose ancestors are believed to include the African wild ass (Beja-Pereira et al., 2004; Rossel et al., 2008). With agricultural and industrial development and mechanization, the donkey has become somewhat redundant as a beast of burden and the global donkey population has notably reduced. China has the largest number of donkeys in Asia, estimated at more than three million in 2017 (Tian et al., 2020). However, as the demand for donkey-hide gelatin products increases, the number of donkeys in China is decreasing. There is now a need to address aspects of donkey breeding in order to preserve the species. Unfortunately, there is little research regarding the reproductive performance of donkeys (Tian et al., 2020; Li Z. et al., 2021). Hence, in this study, comparative transcriptomic and WGCNA were carried out to reveal the developmental characteristics of donkey oocytes, which would provide a theoretical basis for the protection of donkey germplasm.

The current study made an in-depth exploration of gene expression characteristics by thoroughly comparing the similarities and differences in oocyte development during GV–MII between donkeys and cattle, sheep, pigs, and mice (Qian et al., 2019; Wang et al., 2020b; Du et al., 2021; Li et al., 2021a; Li et al., 2021b), Furthermore, the release of the newly assembled donkey reference genome and the update of bioinformatics analysis tools (Wang C. et al., 2020), allowed us to annotate donkey genes more accurately, and to obtaining more refined results. Interestingly, in terms of transcriptome characteristics, we found that the GV and MII phases formed two groups of cells with a clear dividing line (Figure 1). Consistent with the results of previous studies, we found that in the studied species there were far fewer up-DEGs than down-DEGs (Figure 2). This phenomenon may be due to the gene transcription repression that occurs from the GV phase to the MII phase (De La Fuente et al., 2004); previous studies report that maternal mRNA is selectively degraded during development from GV to MII (Su et al., 2007; Zhao et al., 2020). Many studies suggested that non-coding RNAs (ncRNAs) were related to oocyte development (Barragán et al., 2017; Bouckenheimer et al., 2018). Interestingly, a study reported that ncRNAs were related to age and ovarian reserve in human (Barragán et al., 2017). But, the reports of ncRNAs in donkey oocyte development is few.

Unexpectedly, comparative transcriptomic analysis showed that donkey-specific DEGs are involved in the immune response, compared with other species. IL10 is an anti-inflammatory cytokine that is involved in apoptosis (Dhingra et al., 2009), and in this study, we found that it was a donkey specific up-DEG (Figure 4C). It has been reported in the literature that the expression of IL10 in granulosa cells is related to the success rate of IVF (Çavus and Deger, 2020). Moreover, the expression of IL10 attenuates the apoptosis rate of cells (Dhingra et al., 2009). However, few studies reported the role of IL10 in donkey oocytes. Interestingly, donkey specific down-DEGs, such as CXCL10 (Figure 4F), also participated in immune processes. It is reported that, CXCL10 serves as a marker of apoptosis (Sui et al., 2004), and the overexpression of CXCL10 increases the apoptosis of cells (Sui et al., 2006). The up-regulation of anti-apoptotic factors and the down-regulation of pro-apoptotic factors in donkey oocytes may be related to the maintenance of cell survival during oocyte development.

WGCNA was used to find co-expressed gene modules and to explore the relationship between gene networks and the phenotype of interest, as well as the core genes in the network; it is an effective method for analyzing the regulatory relationship of complex gene expression patterns (Pei et al., 2017). There is a consensus that the mechanism of oocyte development and maturation is species-specific (Yamashita et al., 2000). However, WGCNA data in the current study showed similar results for all studied species (Figure 5C). Subsequently, we explored the genes in the modules most related to donkeys and the results suggested that, consistent with the results of comparative transcriptomic analysis, these genes are closely related to the meiotic cell cycle and immune processes. Moreover, we selected 36 hub genes including WEE2, which was consistent with previous reports. Previous research reported that the overexpression of WEE2 is able to postpone the reentry of oocytes into meiosis in both mice and monkeys (Hanna et al., 2010). Furthermore, Wee2-deficient mice exhibit fertilization failure and female infertility (Sang et al., 2018).

## Conclusion

Overall, our research brought new perspectives regarding the development of donkey oocytes. Compared with other species, donkey oocytes express a large number of genes related to RNA metabolism to maintain normal oocyte development during GV–MII. Our study offers a theoretical basis for improving oocyte maturation in the donkey.

## Data Availability

The original contributions presented in the study are included in the article/Supplementary Material, further inquiries can be directed to the corresponding author.

## Supplementary Material

Supplementary Figure S1
**(A)** The evaluation of WGCNA genes of oocyte development during GV–MII in different species. Left panel, analysis of the scale-free fit index for various soft thresholding powers. The blue line represents a scale-free fit index of 0.9. Right panel, analysis of the mean connectivity for various soft-thresholding powers. **(B)** The hierarchical cluster tree shows functional modules distinguished by WGCNA. Bottom part, different colors represent different modules. **(C)** The heat map plot shows the topological overlap matrix (TOM) among 500 randomly selected genes. Light purple color depict a small overlap, and the red color indicates a greater overlap.
