# Supplementary figures and images for: Comparative Transcriptomics Uncover the Uniqueness of Oocyte Development in the Donkey
Source: Front Genet. 2022 Jan 28;13:839207. doi: 10.3389/fgene.2022.839207 (PMC8832878; doi:10.3389/fgene.2022.839207)

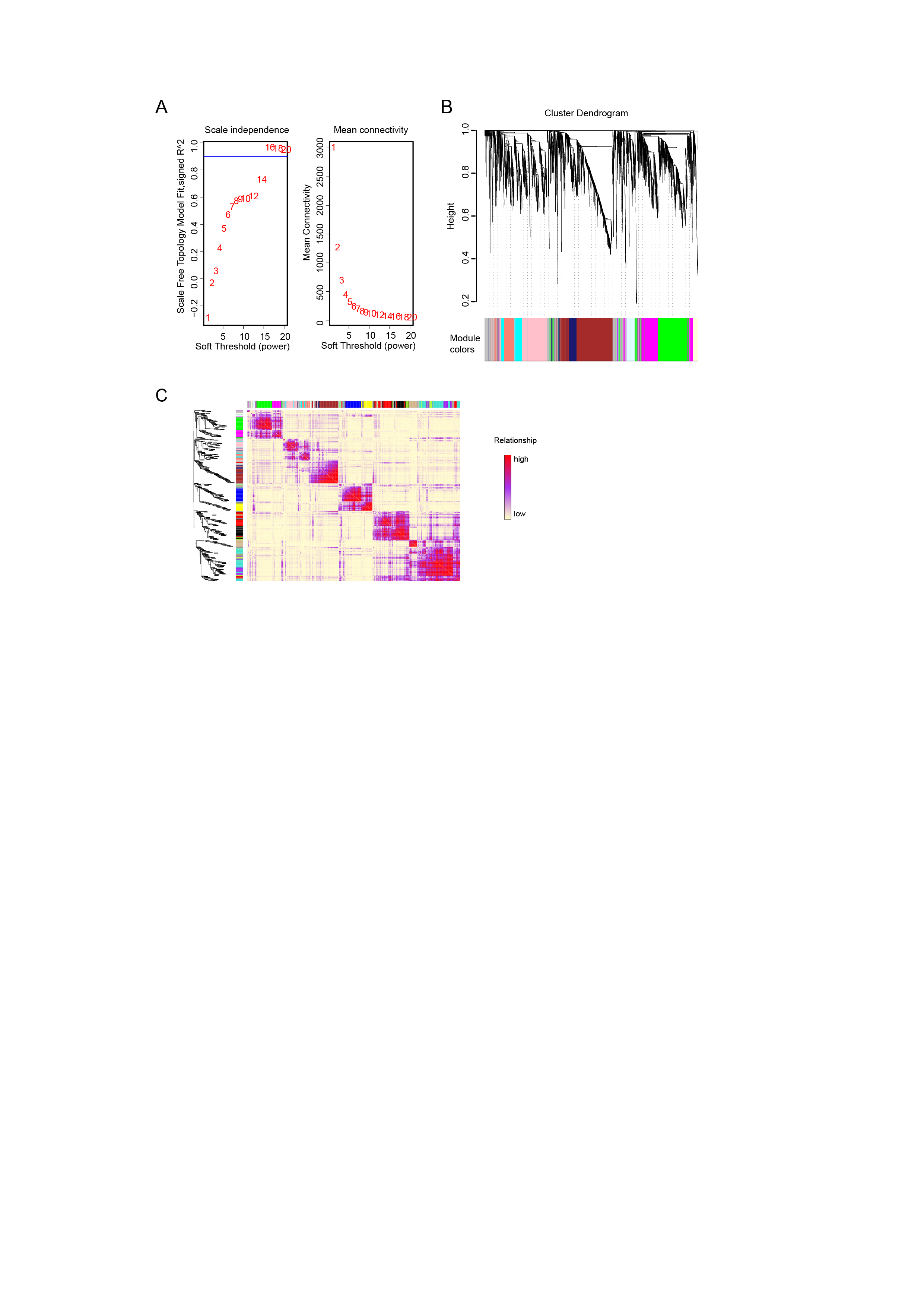

Supplement: Supplementary file 4 [file Image1.TIF]
